# Probiotic *Lactobacilli* ameliorate alcohol-induced hepatic damage *via* gut microbial alteration

**DOI:** 10.3389/fmicb.2022.869250

**Published:** 2022-08-18

**Authors:** Juseok Kim, Seong Woo Ahn, Joon Yong Kim, Tae Woong Whon, Seul Ki Lim, Byung Hee Ryu, Nam Soo Han, Hak-Jong Choi, Seong Woon Roh, Se Hee Lee

**Affiliations:** ^1^Kimchi Functionality Research Group, World Institute of Kimchi, Gwangju, South Korea; ^2^Microbiome Research Team, LISCure Biosciences Inc., Seongnam, South Korea; ^3^Fermentation Regulation Technology Research Group, World Institute of Kimchi, Gwangju, South Korea; ^4^Food Research Division, Food BU, Daesang Corporation Research Institute, Icheon, South Korea; ^5^Department of Food Science and Biotechnology, Brain Korea 21 Center for Bio-Health Industry, Chungbuk National University, Cheongju, South Korea

**Keywords:** alcoholic liver disease, *Lactobacilli*, kimchi, gut microbial community, *Enterococcus feacalis*

## Abstract

Alcoholic liver disease (ALD), which includes fatty liver, cirrhosis, steatosis, fibrosis, and hepatocellular carcinoma, is a global health problem. The probiotic effects of lactic acid bacteria (LAB) are well-known; however, their protective effect against ALD remains unclear. Therefore, in this study, our objective was to assess the protective effects of LAB on ALD. To this end, mice were fed either a normal diet or an alcohol diet for 10 days (to induce ALD) accompanied by vehicle treatment (the NC and AC groups) or kimchi-derived LAB (*Lactiplantibacillus plantarum* DSR J266 and *Levilactobacillus brevis* DSR J301, the AL group; or *Lacticaseibacillus rhamnosus* GG, the AG group). Our results showed that mice in the AC group showed significantly higher serum aspartate aminotransferase and alanine aminotransferase levels than those in the normal diet groups; however, their levels in the AL and AG groups were relatively lower. We also observed that the AL and AG groups showed relatively lower interleukin-6 levels than the AC group. Additionally, AC group showed the accumulation of several fat vesicles in the liver, while the AL and AG groups showed remarkably lower numbers of fat vesicles. The relative abundance of *Enterococcus feacalis*, which showed association with liver injury, significantly increased in the AC group compared with its levels in the normal diet groups. However, the AG group showed a decreased relative abundance in this regard, confirming that LAB exerted an improvement effect on gut microbial community. These findings suggested that *via* gut microbiota alteration, the ingestion of LAB can alleviate the ill effects of alcohol consumption, including inflammation, liver damage, gut dysbiosis, and abnormal intestinal nutrient metabolism.

## Introduction

Alcoholic liver disease (ALD), which is also known as alcohol-related liver disease, and includes fatty liver, cirrhosis, steatosis, fibrosis, and hepatocellular carcinoma (Frazier et al., 2011; Wang et al., 2016), is a common health problem worldwide (Liangpunsakul et al., 2016). Reportedly, chronic alcohol intake increases the risk of ALD owing to the accumulation of reactive oxygen species produced from alcohol metabolism in the liver; this varies among individuals (Galicia-Moreno and Gutierrez-Reyes, 2014; Seitz et al., 2018). It has also been observed that alcohol sensitizes the Kupffer cells residing in the liver; thus, increase inflammation (Szabo et al., 2012; Barnes et al., 2014). Additionally, chronic alcohol consumption causes fat accumulation in the liver, and this can lead to alcoholic fatty liver-related ALD (Thomes et al., 2019).

To treat ALD, three therapeutic strategies have been extensively used. First, psychosocial and behavioral therapies, which involve the process of achieving goals and receiving rewards, have been employed; however, their effectiveness requires more systematic investigation (Addolorato et al., 2016). Second, pharmacological treatments, including the prescription of benzodiazepines, to treat alcohol withdrawal syndrome, have also been used extensively used. However, much caution is required in this regard due to the associated risk of the patients developing hepatic encephalopathy (Leggio and Lee, 2017). The third strategy involves the use of probiotics. Recent studies have shown that probiotic treatment can reduce alcohol-induced intestinal microbial dysbiosis, permeability, bacterial translocation, and endotoxemia, thereby re-establishing a balanced intestinal microbiome and strengthening the gut barrier (Chassaing et al., 2014; Konturek et al., 2018; Hong et al., 2019). Specifically, lactic acid bacteria (LAB), which are well-known probiotic agents that are present in dairy products and fermented foods, reportedly exert beneficial effects, such as immune enhancement and anticarcinogenic effects (Perdigon et al., 2002). Among various LAB species, *Lactobacilli* strains improve liver condition by enhancing innate immunity *via* the regulation of Toll-like receptor expression (Vizoso Pinto et al., 2009; Tsai et al., 2012; Aoki-Yoshida et al., 2016; Khalique et al., 2019). Additionally, the treatment of patients with ALD *via* the administration of probiotics, such as *Lactobacilli* strains, can improve gut microflora, thereby reversing liver damage. Further, One of the foods abundant in LAB is kimchi (Jung et al., 2011; Lee et al., 2015). Kimchi is a traditional fermented food that has been consumed by Koreans for over a thousand years. Therefore, the safety of kimchi-derived LAB has been sufficiently verified over a long period. Since the fermentation environment of kimchi is a low-temperature, high-salt, and low-pH environment, the kimchi microorganisms also have a wide growth spectrum range and can proliferate in even in harsh environments, such as the intestinal tract of animals and exhibit functionality. In particular, it has been reported that bacteriocin produced by kimchi LAB is stable to heat and low pH conditions (Choi et al., 2000; Cheigh et al., 2002; Ahn et al., 2012; Seo et al., 2014). In previous studies, the effects of kimchi LAB, including immunomodulatory effects, atopic dermatitis, and hypercholesterolemia improvement effects, antioxidant activity, anti-obesity effects, and pathogen infection prevention, were also verified (Lim and Choi, 2019; Lee et al., 2020). Additionally, kimchi LAB with these functions can be added to foods, such as kimchi again, to facilitate their consumption. Even though the probiotic effects of kimchi-derived LAB are well-known (Lee et al., 2020), their protective effects against ALD are still unclear. Therefore, in this study, we aimed to confirm the effects of kimchi-derived LAB on ALD prevention and subsequent recovery using a mouse model of ALD.

## Materials and methods

### Isolation, screening, and preparation of kimchi-derived lactic acid bacteria

The kimchi sample used in this study (Daesang Corporation, Seoul, Republic of Korea) was serially diluted in de Man-Rogosa-Sharpe broth medium (MRS; Becton, Dickinson and Company, Sparks, MD, United States). Thereafter, aliquots of each serial dilution were spread on MRS agar plates, which were then incubated at 30^°^C for 2 days. The colonies thus obtained were analyzed using 16S rRNA gene sequencing as previously described (Lim et al., 2017). Subsequently, *Lactiplantibacillus* (*Lp*.) *plantarum* DSR J266 and *Levilactobacillus* (*Lv*.) *brevis* DSR J301 isolated from the kimchi were cultured in MRS broth at 30^°^C for 2 days and harvested *via* centrifugation at 8,000 *g* and 4^°^C for 15 min. Thereafter, the harvested cells were washed three times with phosphate-buffered saline (PBS), followed by the resuspension of the two strains in PBS to a final concentration of 5 × 10^8^ colony forming units (CFU)/100 μL and equal mixing for administration *via* gavage. *Lacticaseibacillus* (*Lb.*) *rhamnosus* GG was isolated and prepared using the same process, and the final concentration was adjusted to 1 × 10^9^ CFU/200 μL for administration *via* gavage.

### Animal studies

**FIGURE 1:**
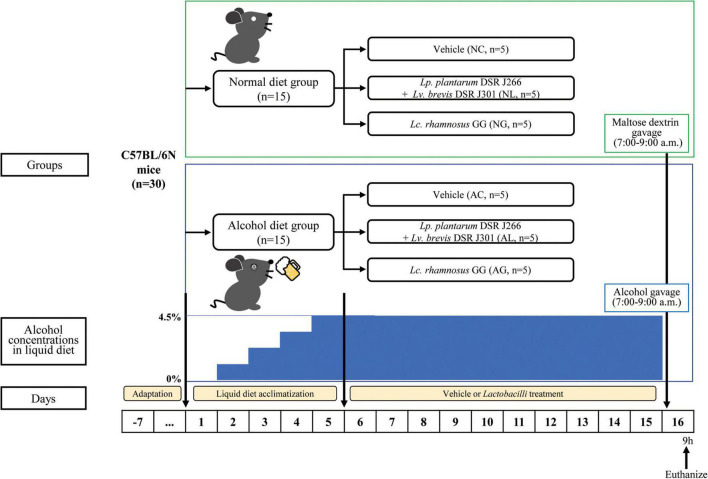
Experimental procedure. All C57BL/6N mice are initially fed the Lieber-DeCarli diet *ad libitum* for 5 d to acclimatize them to a liquid diet and tube feeding. Before the acclimatization period, the mice were randomly divided into the following two diet groups (*n* = 15 per group): normal diet group (mice were fed Liber-DeCarli control diet) and alcohol diet group (mice were fed Liber-DeCarli diet containing alcohol). The alcohol concentration in the alcohol diet group was gradually increased to 4.5% for 5 days to enable animals adapt to the alcohol diet. The two groups were further randomly subdivided into each of the following three LAB-treatment groups, resulting in a total of six groups (*n* = 5 per group): normal diet and vehicle (NC); normal diet and *Lp*. *plantarum* DSR J266 + *Lv*. *brevis* DSR J301 (NL); normal diet and *Lb*. *rhamnosus* GG (NG); alcohol diet and vehicle (AC); alcohol diet and *Lp*. *plantarum* DSR J266 + *Lv*. *brevis* DSR J301 (AL); and alcohol diet and *Lb*. *rhamnosus* GG (AG). Mice in the alcohol diet groups (AC, AL, and AG) were allowed free access to the Lieber-DeCarli diet containing 4.5% (vol/vol) ethanol for 10 days, while those in the normal diet groups (NC, NL, and NG) were pair-fed with the isocaloric control diet replacing ethanol with maltose dextrin. On day 16, ethanol-fed and pair-fed mice were gavaged in the early morning with a single dose of ethanol (5 g kg^– 1^ body weight) or isocaloric maltose dextrin, respectively, and 9 h later, the mice were euthanized. Next, blood, liver tissue, and fecal samples were collected for subsequent analysis.

Six-week-old wild-type male C57BL/6N mice were purchased from Orient Bio Inc. (Seongnam, Republic of Korea). The animals were housed (2–3 mice per individually ventilated cage) in a pathogen-free animal facility at the World Institute of Kimchi (Gwangju, Republic of Korea, Republic of Korea), and maintained at 22 ± 2^°^C and 55 ± 5% relative humidity under a 12-h/12-h light-dark cycle. After the animals were acclimatized for 1 week before the experiments, they were randomly divided into the following six diet groups (*n* = 5 per group, Figure 1): normal diet and vehicle (NC); normal diet and *Lp*. *plantarum* DSR J266 + *Lv*. *brevis* DSR J301 (NL); normal diet and *Lb. rhamnosus* GG (NG); alcohol diet and vehicle (AC); alcohol diet and *Lp*. *plantarum* DSR J266 + *Lv*. *brevis* DSR J301 (AL); and alcohol diet and *Lb. rhamnosus* GG (AG). Mice were fed a nutritionally adequate Lieber-DeCarli liquid diet (LDC; Doo Yeol Biotech, Seoul, Republic of Korea) throughout the study period (Lieber and DeCarli, 1982). Alcohol liver injury was induced in mice using the modified NIAAA model (Bertola et al., 2013; Guo et al., 2018). Further, mice in the NC, NL, and NG groups were fed the LDC liquid diet for 15 days, whereas those in the AC, AL, and AG groups were fed the LDC liquid diet containing alcohol. During the first 5 days, the alcohol concentration in the diet was gradually increased to 4.5%. This was to allow the animals adapt to the alcohol diet. Subsequently, the LDC liquid diet containing 4.5% alcohol was fed to the mice in the AC, AL, and AG groups for the remaining 10 days. During the same period, mice in the NL and AL groups were gavaged daily with *Lp. plantarum* DSR J266 and *Lv. brevis* DSR J301, while those in the NG and AG groups were gavaged daily with *Lb. rhamnosus* GG. Furthermore, mice in the AC and NC groups were gavaged daily with PBS (vehicle). The gavage was always performed in the morning (9–10 am), and in the afternoon, the feeding tubes and LDC liquid diet were replaced with new ones (4–5 pm). The calories in the normal and alcohol liquid diets were adjusted using maltose dextrin (Guo et al., 2018). On day 16, mice in the alcohol diet groups (AC, AL, and AG groups) were administered a single alcohol binge (5 g kg^–1^ body weight, 20% alcohol), whereas mice in the normal diet groups (NC, NL, and NG) were gavaged with isocaloric maltose dextrin. After 9 h, when serum levels of alanine aminotransferase (ALT), aspartate aminotransferase (AST), and inflammatory cytokines peaked (Bertola et al., 2013), the all mice were euthanized and their blood, tissue, and fecal samples were collected.

### Serological analysis

Serum was separated from the collected blood samples *via* centrifugation at 3,000 *g* for 15 min at 4°C. Thereafter, serum AST and ALT levels were determined using a FUJI DRI-CHEM NX500iVC biochemical analyzer with their respective FUJI DRI-CHEM slide kits (FujiFilm Corporation, Tokyo, Japan). In addition, serum levels of inflammatory cytokines, including interleukin (IL)-6, IL-10, monocyte chemotactic protein (MCP)-1, interferon (IFN)-γ, tumor necrosis factor (TNF)-α, and IL-12p70, were detected using the BD™ Cytometric Bead Array kit and the BD FACSCanto II flow cytometry system (BD Biosciences, San Jose, CA, United States).

### Histological analysis

The collected liver tissue samples were fixed in 10% formalin at 25^°^C for 24 h followed by embedding in paraffin, sectioning, mounting on glass slides, and staining with hematoxylin and eosin (H&E). Thereafter, representative photomicrographs were captured using an Olympus BX41 microscope (Olympus, Tokyo, Japan) at 100 × magnification. Further, to analyze the captured images, Olympus CellSens Standard software version 1.6 was used.

### Microbiome analysis

The total genomic DNA corresponding to the collected fecal samples was extracted *via* mechanical and chemical lysis, and purified using a Mo Bio PowerSoil DNA Isolation Kit (Mo Bio Laboratories, Carlsbad, CA, United States) according to the manufacturer’s instructions. Thereafter, the quality of the DNA was measured *via* PicoGreen assay, performed using a NanoDrop UV spectrophotometer (Thermo Fisher Scientific, Waltham, MA, United States). Next, genomic DNA was amplified using primers targeting the V3 to V4 hypervariable regions of the 16S rRNA gene (S-D-Bact-0341-b-S-17, 5′-CCTACGGGNGGCWGCAG-3′; S-D-Bact-0785-a-A-21, 5′-GACTACHVGGGTATCTAATCC-3′) (Herlemann et al., 2011; Klindworth et al., 2013). Paired-end sequencing was further performed by Macrogen Inc. (Seoul, Republic of Korea), using an Illumina MiSeq platform (Illumina Inc., San Diego, CA, United States). The generated sequencing reads were analyzed using QIIME2 software version 2019.10 pipeline (Bolyen et al., 2019), which utilizes 16S rDNA sequences in the Silva v132 database (Quast et al., 2013; Yilmaz et al., 2014), and to predict the functional profiles of the mouse gut microbial community based on the 16S rRNA gene dataset, PICRUSt software (Langille et al., 2013) was used. The 16S rRNA gene sequencing data were deposited in the National Center for Biotechnology Information (NCBI) GenBank under accession no. SRX12860986.

### Statistical analysis

All the results for qualitative data were presented as the mean ± standard error of mean (SEM). The serological characteristics that differed significantly between the diet groups were determined using one-way ANOVA and Tukey’s HSD *post-hoc* test (*p* ≤ 0.001). The level of significance was set at *p < 0.05, ^**^p < 0.01, ^***^p < 0.001, ^****^p < 0.0001.

### Ethical statement

All animal procedures were performed according to the National Institute of Health Guidelines for the Humane Treatment of Animals, with approval from the Institutional Animal Care and Use Committee of the World Institute of Kimchi (WIKIM IACUC 201936). The animals were sacrificed *via* forced CO_2_ inhalation and all efforts were made to minimize suffering.

## Results

### Effect of lactic acid bacteria administration on serum aminotransferase, aminotransferase, and inflammatory cytokine levels

**FIGURE 2:**
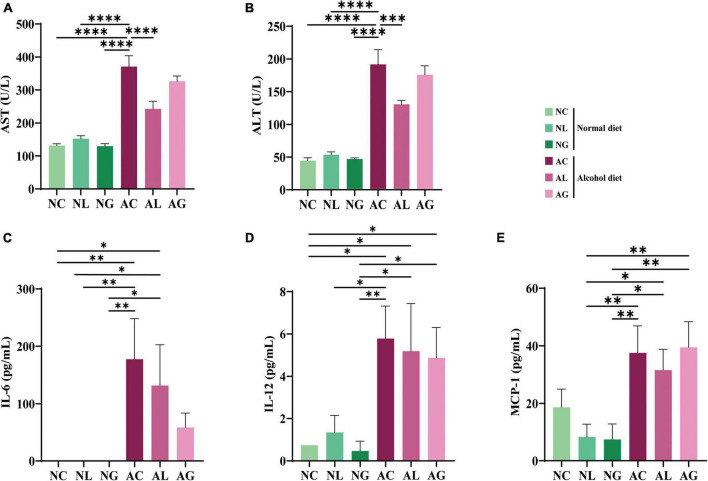
Serum levels of ALT, AST, and inflammatory cytokines in mice fed with normal and alcohol diet. **(A)** ALT, **(B)** AST, and inflammatory cytokines **(C)** IL-6, **(D)** IL-12, and **(E)** MCP-1. The data were analyzed by performing ordinary one-way ANOVA followed by Fisher’s LSD test. Data are expressed as mean ± standard error of mean (SEM), *p < 0.05, ^**^p < 0.01, ^***^p < 0.001, ^****^p < 0.0001. AST, aspartate transaminase; ALT, alanine aminotransferase; IL, interleukin; MCP, monocyte chemotactic protein; NC, normal diet group; NL, normal diet and *Lactiplantibacillus* (*Lp*.) *plantarum* DSR J266 + *Levilactobacillus* (*Lv*.) *brevis* DSR J266 group; NG, normal diet and *Lacticaseibacillus* (*Lb*.) *rhamnosus* GG group; AC, alcohol diet group; AL, alcohol diet and *Lp*. *plantarum* DSR J266 + *Lv*. *brevis* DSR J301 group; AG, alcohol diet and *Lb. rhamnosus* GG group; LSD, least significant difference.

To investigate the effect of LAB strains (Figures 2A,B) on the degree of alcohol-induced liver damage, the serum ALT and AST levels of mice in the different treatment groups were measured (Bertola et al., 2013; Torruellas et al., 2014). Thus, we observed that in the alcohol-fed mice, AST levels were approximately 2-fold ALT levels. Previous studies have reported that an AST/ALT ratio of 1.5 or 2.0 indicates alcoholic hepatitis (Cohen and Kaplan, 1979; Alves et al., 1981; Correia et al., 1981). Further, compared with the normal diet groups, AST and ALT levels were significantly higher in the alcohol diet groups (Figures 2A,B; ANOVA, *p* < 0.05); this is consistent with the results of a previous study (Bertola et al., 2013). We also observed that the normal diet groups showed no significant differences with respect to AST and ALT levels. The differences in AST and ALT levels among the alcohol diet groups could be attributed to the presence, absence, or type of LAB ingested. Further, compared with the AC group, AST and ALT levels in the AL group were significantly decreased (ANOVA, *p* < 0.05). These results indicated that LAB treatment in the normal diet groups did not affect AST and ALT levels, whereas LAB treatment in the alcohol diet groups decreased AST and ALT levels.

To further investigate the effects of LAB treatment on ALD mice, the levels of inflammatory cytokines (IL-6, IL-10, MCP-1, IFN-γ, TNF-α, and IL-12) in sera samples from mice in the different treatment groups were analyzed. Thus, IL-6, IL-12, and MCP-1 were identified as the main inflammatory cytokines in the samples (Figures 2C–E); IL-10, IFN-γ, and TNF-α were not detected in any of the samples. Further, IL-6 was not detected in samples from the normal diet groups, but was observed in samples from the alcohol diet-fed mice (Figure 2C). The average IL-6 concentration corresponding to the AC group was higher than that corresponding to the AL and AG groups, indicating a decrease in inflammatory cytokine levels following LAB treatment. Reportedly, IL-6 is a representative inflammatory cytokine, and its level is elevated in animal models of alcohol-related liver injury (Latvala et al., 2005). Furthermore, the AL and AG groups displayed relatively low IL-6 levels, suggesting that LAB treatment protected these mice from alcohol-induced hepatocyte damage. Moreover, the levels of proinflammatory cytokines, IL-12 and MCP-1, were relatively higher in the alcohol diet groups than in the normal diet groups (Figures 2D,E), indicating that IL-12 and MCP-1 may be closely involved in the progression of alcoholic liver injury.

### Effects of lactic acid bacteria administration on hepatic histology

**FIGURE 3:**
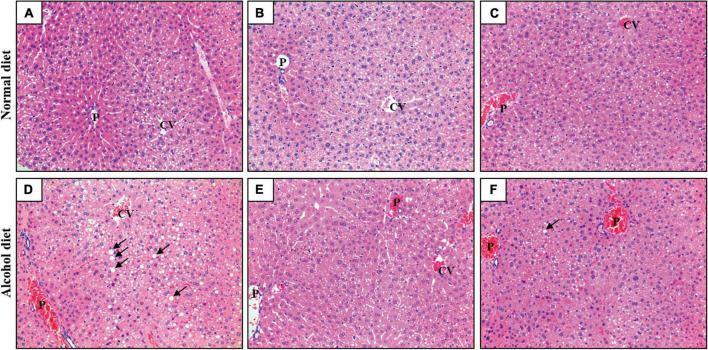
Hematoxylin and eosin staining of liver sections (×200). **(A)** NC, normal diet group; **(B)** NL, normal diet and *Lactiplantibacillus* (*Lp*.) *plantarum* DSR J266 + *Levilactobacillus* (*Lv*.) *brevis* DSR J266 group; **(C)** NG, normal diet and *Lacticaseibacillus* (*Lb*.) *rhamnosus* GG group; **(D)** AC, alcohol diet group; **(E)** AL, alcohol diet and *Lp*. *plantarum* DSR J266 + *Lv*. *brevis* DSR J301 group; **(F)** AG, alcohol diet and *Lb. rhamnosus* GG group. Lipid droplets are indicated using black arrows. CV, central vein; P, portal vein.

Liver tissue samples from mice in the different treatment groups were subjected to H&E staining to confirm ALD induction and the accumulation of neutral lipid (triglyceride) droplets (Figure 3). Mice in the normal diet groups (the NC, NL, and NG groups) showed healthy liver tissues, whereas those in the alcohol diet groups showed fat accumulation in their livers. However, the AL and AG groups exhibited relatively less liver fat accumulation than the AC group. These results suggested that although LAB intake did not affect fat accumulation in the livers of mice that consumed a normal diet, it prevented fat accumulation in the hepatic tissues of alcohol diet-fed mice (Cai et al., 2019; Hsieh et al., 2021).

### Effect of lactic acid bacteria administration on the gut bacterial community

**FIGURE 4:**
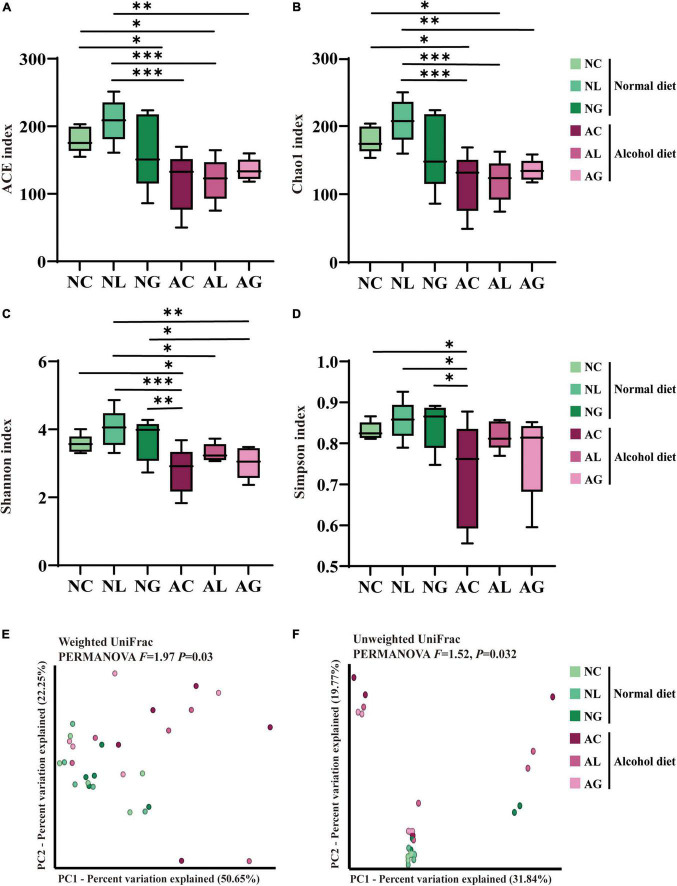
Effect of LAB intake on gut microbial alpha- and beta-diversity in normal and alcohol diet-fed mice. The **(A)** ACE and **(B)** Chao1 indices indicate ASV richness, while the **(C)** Shannon and **(D)** Simpson indices indicate ASV evenness in the mice fecal samples. The boxes represent the interquartile range between the 25th and 75th percentile, and the horizontal line inside the box denotes the median. The data were analyzed by performing ordinary one-way ANOVA followed by Fisher’s LSD test. Data are expressed as mean ± standard error of mean (SEM), *p < 0.05, ^**^p < 0.01, ^***^p < 0.001. The principal coordinates analysis (PCoA) plot was constructed using **(E)** Weighted and **(F)** Unweighted UniFrac distance matrices. The data were analyzed *via* PERMANOVA using 999 permutations. NC, normal diet group; NL, normal diet and *Lactiplantibacillus* (*Lp*.) *plantarum* DSR J266 + *Levilactobacillus* (*Lv*.) *brevis* DSR J266 group; NG, normal diet and *Lacticaseibacillus* (*Lb*.) *rhamnosus* GG group; AC, alcohol diet group; AL, alcohol diet and *Lp*. *plantarum* DSR J266 + *Lv*. *brevis* DSR J301 group; AG, alcohol diet, and *Lb. rhamnosus* GG group; LSD, least significant difference.

The bacterial 16S rRNA gene amplicon obtained from mice fecal samples was sequenced to confirm differences in gut bacterial community composition owing to alcoholic liver injury and LAB intake. A total of 3,776,969 sequencing reads for bacterial 16S rRNA genes were generated from the 30 collected fecal samples. After removing low-quality sequences, noise, unmerged sequences, chimeras, and singletons, 2,240,092 high-quality bacterial reads were obtained, with an average of 74,670 reads per sample (Supplementary Table 1). Further, we observed that the bacterial alpha-diversity indices of the alcohol diet groups was lower than those of the normal diet groups, based on the ACE/Chao1 indices (Figures 4A,B) and Shannon-Simpson indices (Figures 4C,D) obtained (Fairfield and Schnabl, 2020). Nevertheless, the decrease in bacterial diversity in the AC group was alleviated by LAB treatment based on the diversity indices obtained for the AL and AG groups. These results suggested that alcohol consumption reduced the microbial diversity in the intestine, which however, was restored by LAB treatment. The beta-diversity of the bacterial community was analyzed using weighted and unweighted UniFrac distances (Figures 4E,F), which revealed a significant difference between the normal and alcohol diet groups (PERMANOVA, *p* < 0.05). In addition, the distances between normal diet group plots were similar, whereas the alcohol diet group plots were widely separated, confirming that the alcohol diet induced greater differences in microbial diversity between the treatment groups than the normal diet.

**FIGURE 5:**
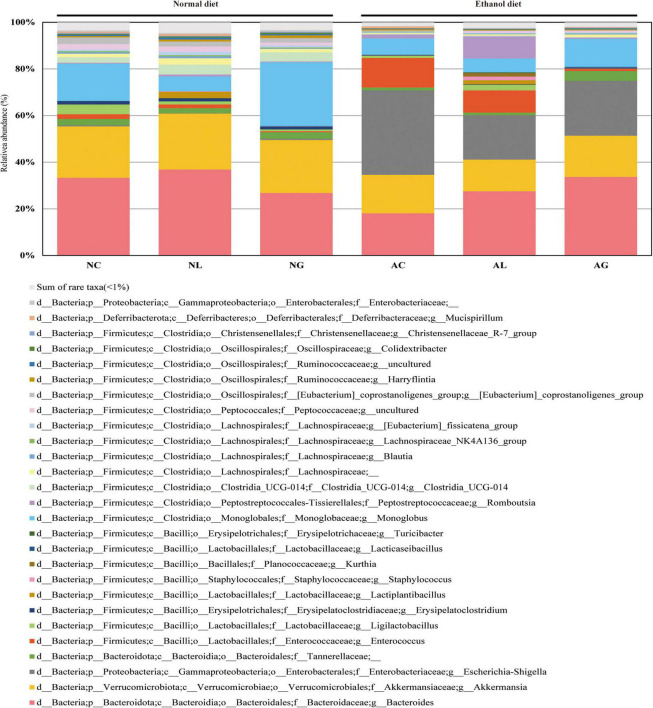
Effect of administering kimchi-derived LAB on the relative abundance of gut microbiota in normal and alcohol diet-fed mice. The relative bacterial mean abundance at the genus level was calculated taking into consideration the mean value of the relative phylotypic compositions of respective fecal samples. The sum of rare taxa consisted of genera showing percentage of reads < 0.5% considering the total reads from all subjects.

**FIGURE 6:**
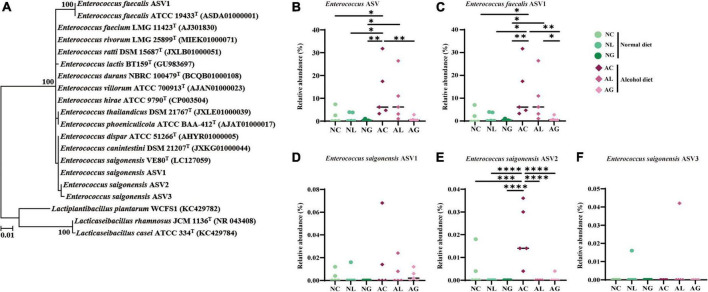
Phylogenetic analysis and relative abundance of the amplicon sequence variants (ASVs) of *Enterococcus*. The ASVs assigned to *Enterococcus* originated from bacterial 16S rRNA gene sequence data of mice fed normal and alcohol diets. **(A)** Phylogenetic tree based on the 16S rRNA gene sequences and reconstructed using the maximum likelihood algorithm, indicating the taxonomic positions of the ASVs. Further, the 16S rRNA gene sequences of the validated *Enterococcus* species were included. The bootstrap values are shown on the nodes as percentages corresponding to only 1,000 replicates with values over 70%. *Lactiplantibacillus plantarum* WCFS1, *Lacticaseibacillus rhamnosus* JCM 1136*^T^*, *Lactobacillus casei* ATCC 334*^T^* were used as the outgroup. The scale bar equals 0.01 changes per nucleotide position. Relative abundance of **(B)** total *Enterococcus* ASV, **(C)** *Enterococcus feacalis* ASV1, **(D)** *Enterococcus saigonensis* ASV1, **(E)** *Enterococcus saigonensis* ASV2, and **(F)** *Enterococcus saigonensis* ASV3. The data were analyzed by performing ordinary one-way ANOVA followed by Fisher’s LSD test. *p < 0.05, ^**^p < 0.01, ^***^p < 0.001, ^****^p < 0.0001. NC, normal diet group; NL, normal diet and *Lactiplantibacillus* (*Lp*.) *plantarum* DSR J266 + *Levilactobacillus* (*Lv*.) *brevis* DSR J266 group; NG, normal diet and *Lacticaseibacillus* (*Lb*.) *rhamnosus* GG group; AC, alcohol diet group; AL, alcohol diet and *Lp*. *plantarum* DSR J266 + *Lv*. *brevis* DSR J301 group; AG, alcohol diet and *Lb. rhamnosus* GG group; LSD, least significant difference.

To further investigate the effect of LAB intake on alcohol-induced liver injury in mice, gut microbiome composition was analyzed. Thus, we observed that the microbiomes of the alcohol and normal diet groups differed substantially (Figure 5 and Supplementary Figure 1). The abundances of the genera *Akkermansia* and *Monoglobus* were relatively high in the normal diet group, but low in the alcohol diet groups. However, the abundances of the genera *Escherichia*-*Shigella* and *Enterococcus* were relatively higher in the alcohol diet groups compared with their abundances in the normal diet groups (Fairfield and Schnabl, 2020). We also observed that the abundances of the genus *Escherichia*-*Shigella* and *Enterococcus* differed depending on the LAB treatment. Specifically, *Escherichia*-*Shigella* and *Enterococcus* were the most dominant genera in the AC group, but showed decreased abundances in the AL and AG groups. Reportedly, species of the family *Enterobacteriaceae*, to which the genus *Escherichia*-*Shigella* belongs, are prevalent in the intestine of patients with alcoholic cirrhosis (Chen et al., 2011; Bajaj et al., 2012; Tuomisto et al., 2014). Most of the sequences classified as the genus *Enterococcus* were confirmed to be from *Enterococcus faecalis* ASV1 (Figure 6). Further, *Enterococcus faecalis* ASV1 was present at a very low frequency in the samples from the normal diet groups; however, it was significantly increased in the AC group (Figure 6), and in the AG group *Enterococcus faecalis* ASV1 was significantly decreased to the level observed for the normal diet groups.

### Effect of lactic acid bacteria administration on the predicted functions of abundant gut bacteria

**FIGURE 7:**
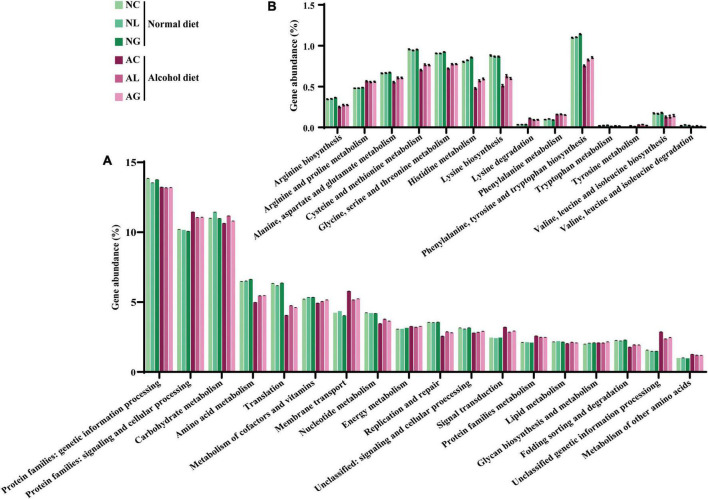
Predicted gene abundance profiles of representative KEGG BRITE functional categories. Data were obtained at **(A)** Secondary and **(B)** tertiary (amino acid metabolism) levels. Data are presented as the mean ± standard deviation. NC, normal diet group; NL, normal diet and *Lactiplantibacillus* (*Lp*.) *plantarum* DSR J266 + *Levilactobacillus* (*Lv*.) *brevis* DSR J266 group; NG, normal diet and *Lacticaseibacillus* (*Lb*.) *rhamnosus* GG group; AC, alcohol diet group; AL, alcohol diet and *Lp*. *plantarum* DSR J266 + *Lv*. *brevis* DSR J301 group; AG, alcohol diet, and *Lb. rhamnosus* GG group.

**FIGURE 8:**
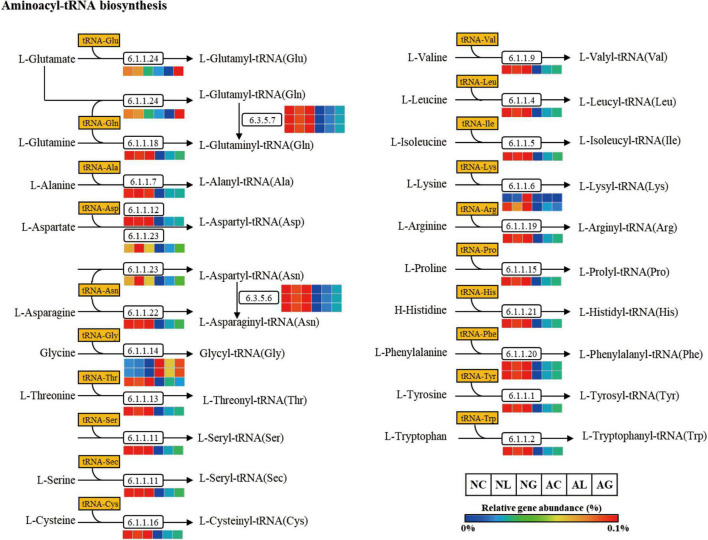
Aminoacyl-tRNA biosynthesis pathway predicted from an OTU table using the PICRUSt algorithm. The pathway was predicted making use of references from the KEGG BRITE functional database, modified from http://www.genome.jp/kegg/pathway/map/map00970.html. NC, normal diet group; NL, normal diet and *Lactiplantibacillus* (*Lp*.) *plantarum* DSR J266 + *Levilactobacillus* (*Lv*.) *brevis* DSR J266 group; NG, normal diet and *Lacticaseibacillus* (*Lb*.) *rhamnosus* GG group; AC, alcohol diet group; AL, alcohol diet and *Lp*. *plantarum* DSR J266 + *Lv*. *brevis* DSR J301 group; AG, alcohol diet and *Lb. rhamnosus* GG group; OUT, operational taxonomic unit.

**FIGURE 9:**
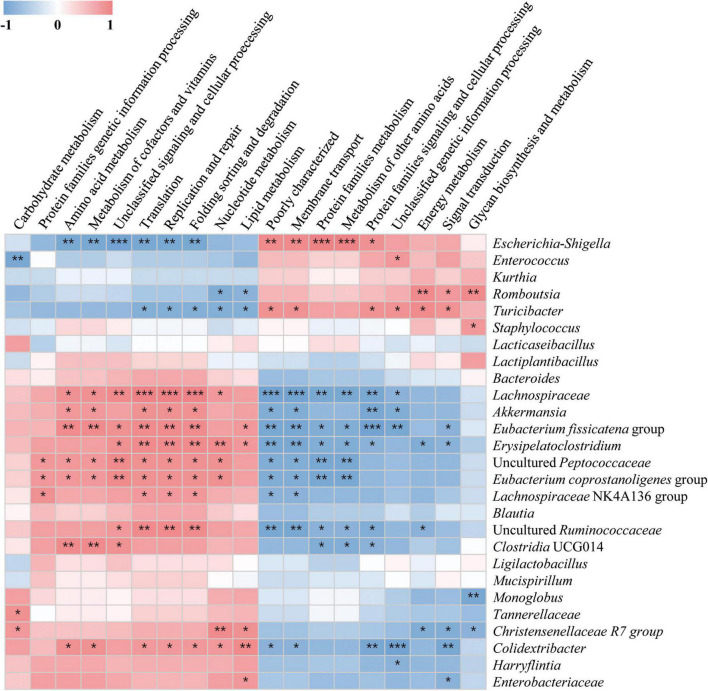
Associations between altered gut microbial community and predicted KEGG BRITE functional categories. The associations were assessed at the secondary level by performing Spearman’s correlation analysis involving microbial community abundance and functional variables. The intermediate heat map corresponds to the Spearman correlation coefficient, r, with values between −1 and 1. r < 0 indicates a negative correlation, while r > 0 indicates a positive correlation. *p < 0.05, ** *p* < 0.01, and ***p < 0.001.

To investigate differences in microbial metabolism following the LAB treatment of normal and alcohol diet fed mice, metabolic functions were predicted using PICRUSt based on the obtained 16S rRNA gene sequencing data (Figures 7A,B) and KEGG BRITE metabolic categories. Thus, we observed substantial differences in gene abundance for each metabolic category according to the treatment diets. Further, the gene abundance according to the type of LAB administered was comparable among the normal diet groups, but considerably differed among the alcohol diet groups. Specifically, genes associated with protein families (i.e., signaling and cellular processes, membrane transport, and signal transduction) were present in relatively high proportions in the alcohol diet groups than in the normal diet groups. In contrast, genes associated with amino acid metabolism, translation, nucleotide metabolism, and replication and repair categories were present in relatively low proportions in the alcohol diet groups compared with the normal diet groups. Additionally, genes associated with the metabolism and biosynthesis of alanine, aspartate, glutamate, arginine, cysteine, methionine, glycine, serine, threonine, histidine, lysine, phenylalanine, tyrosine, tryptophan, valine, leucine, and isoleucine, were present in relatively lower proportions in the alcohol diet groups than in the normal diet groups (Figure 7B). The alteration of intestinal microflora by alcohol administration also affected the abundance of aminoacyl-tRNA biosynthesis-related genes (Figure 8). Specifically, the abundance of genes related to aminoacyl-tRNA biosynthesis was similar among the normal diet groups, but decreased in the alcohol diet groups; however, their abundance recovered slightly after LAB treatment. Decreased expression of aminoacyl-tRNA biosynthesis-related genes may affect amino acid synthesis by intestinal bacteria, but can be recovered *via* LAB treatment. Previous studies have confirmed that acute ALD can be alleviated by regulating the pathways related to aminoacyl-tRNA biosynthesis (Yi et al., 2021). Positive and negative correlation analyses between gut bacterial community composition and the predicted metabolic categories were performed to identify the bacteria involved in microbial metabolism (Figure 9). Thus, we observed that the frequency of amino acid metabolism genes showed negative correlation with *Escherichia-Shigella* and *Enterococcus*, which was relatively predominant in the alcohol group. However, *Akkermansia*, which was dominant in the normal diet group, showed a significant positive correlation.

## Discussion

In this present study, we administered probiotic *Lactobacilli* strains, such as *Lp. plantarum* DSR J266, *Lv. brevis* DSR J301, and *Lb*. *rhamnosus* GG to mice with induced ALD and mice fed a normal diet, and thereafter, verified the amelioration of alcohol-induced liver damage and inflammation, as well as the changes in the composition of the intestinal microbial community of the mice. Compared with the normal diet- or the normal diet + LAB-fed mice, alcohol diet-fed mice showed significant increases in serum AST, ALT, and inflammatory cytokines (IL-6, IL-12, and MCP- 1) levels. These results indicated that ALD was successfully induced using the NIAAA model. Reportedly, IL-6 may protect against hepatocyte apoptosis and participate in mitochondrial DNA repair in chronic ALD (Sheron et al., 1991; Hong et al., 2002). It has also been observed that IL-6 knockout mice subjected to chronic alcohol consumption exhibit lipid peroxidation, liver fat accumulation, and mitochondrial DNA damage, which can be prevented by administering recombinant IL-6 (Hong et al., 2002; El-Assal et al., 2004; Zhang et al., 2010). These results indicated that increased IL-6 levels protect hepatocytes in a mouse model of chronic ALD. Further, IL-12 and MCP-1 reportedly regulate macrophage activation, pro-inflammatory responses, and hepatic steatosis in ALD (Tung et al., 2010; Mandrekar et al., 2011). According to the results of previous studies, chronic alcohol intake reduces the adenosine-monophosphate-activated protein kinase (AMPK) activity involved in lipid metabolism in various tissues, including the liver. However, the intake of LAB increases the expression of AMPK related to triglyceride synthesis. Thus, it has been shown that LAB reduce alcohol-induced fat accumulation and alleviate ALD (Zhang et al., 2015). Additionally, the administration of LAB induces a decrease in hepatic triglyceride concentration caused by non-ALD (NALD) as well as ALD (Kwon et al., 2019). These previous findings indicate that LAB treatment inhibits the production of IL-6, IL-12, and MCP-1, as well as the accumulation of lipid droplets that cause alcoholic liver damage, suggesting they could play a role in alleviating ALD.

The modulation of intestinal microbiota using *Lactobacilli* strains is an appropriate strategy for reducing pathogenic bacteria, such as *Enterococcus faecalis* and *Escherichia coli*. According to recent studies, *Enterococcus faecalis* colonization in the intestinal tract acts as a key promoter in liver carcinogenesis (Iida et al., 2021; Ray, 2021). When intestinal permeability increases due to alcohol intake, *Enterococcus faecalis* causes damage to hepatocytes (Llorente et al., 2017; Duan et al., 2019; Iida et al., 2021); however, the ALD that ensues can be prevented by bacteriophages against *Enterococcus faecalis* (Duan et al., 2019; Çolakoǧlu et al., 2020). Further, the ingestion of *Lb*. *rhamnosus* GG can also help prevent alcoholic liver damage by significantly inhibiting the number of *Enterococcus faecalis* that have hepatocyte-targeted cytolysin. Furthermore, previous studies have shown decreased gene expression of gut barrier hyperpermeability induced by *Escherichia coli* or LPS is restored by *Lb. rhamnosus*. Similar observations have also been made following treatment with other *Lactobacilli* strains. *Lactobacilli* also increases the expression of immune genes related to gut barrier integrity (Blackwood et al., 2017; Lepine et al., 2018; Lee et al., 2020). In this study, we observed that alcohol administration not only changed the composition of the intestinal microbiome, but also brought about changes in metabolic process, such as amino acid metabolism. It is well known that the amino acid content in the intestine decreases rapidly after alcohol intake (Xie et al., 2013).

The gene prediction results, which showed significantly lower amino acid biosynthesis-related genes, suggested that intestinal microbial dysbiosis caused by alcohol consumption may have affected mouse intestinal amino acid content. Thus, correlation analysis could be performed to confirm that the key microbiota associated with intestinal dysbiosis and their metabolisms following chronic alcohol administration, or identify the metabolic processes that were enhanced owing by the administration of lactobacilli to restore the intestine. Thus, a correlation was observed between the presence of *Enterococcus faecalis* in the intestinal microflora and alcoholic liver injury, and reducing *Enterococcus faecalis* abundance was identified as a key strategy for inhibiting alcoholic liver injury. Our results also indicated that the number of *Enterococcus faecalis* could be significantly controlled by the administration of *Lactobacilli* strains. It is well known that microbiome changes in the intestinal tract can lead to changes in microbial metabolism and function, and microorganisms, such as *Akkermansia* influence intestinal amino acid production (Liu et al., 2021). However, ALD-induced microbial dysbiosis led to the dominance of *Escherichia-Shigella* and *Enterococcus* (Liu et al., 2004; Bajaj et al., 2012; Tuomisto et al., 2014), suggesting a decrease in intestinal amino acid metabolism, which can affect not only the intestinal tract and serum, but also muscle and brain metabolism (Kirpich et al., 2015). Additionally, the dominance of *Escherichia*-*Shigella* and *Enterococcus* have a negative correlation with lipid, nucleotide, and vitamin metabolism, suggesting that they can adversely affect overall intestinal metabolism (Zhu et al., 2021). It has also been reported that intestinal microbial dysbiosis induced by alcohol consumption may lead to changes in microbial primary metabolism, suggesting that it may also affect the intestinal tract as well as surrounding organs, including the liver (Zhong and Zhou, 2014; Bajaj, 2019; Fairfield and Schnabl, 2020). Additionally, the decreased abundance of genes related to protein synthesis (aminoacyl-tRNA biosynthesis-related genes) and amino acid metabolism caused by alcohol consumption can affect the ability of intestinal microorganisms to metabolize amino acids in the intestinal tract. Further, chronic alcohol consumption-induced intestinal microbial dysbiosis can rapidly lead to a decrease in the levels of metabolites, including amino acids, fatty acids, steroids, lipids, carnitine, and bile acids, in the intestine. It has also been observed that decreased amino acid levels, as well as intestinal microbial dysbiosis, can interfere with gut microbiota-host co-metabolism, leading to adverse effects on organs, including the liver (Zhong and Zhou, 2014).

In summary, our results suggest that the consumption of *Lactobacillus* strains can directly alleviate ALD symptoms, including a reduction in inflammatory cytokines, inhibition of fatty liver, and restoration of intestinal dysbiosis. In addition, it can indirectly help to alleviate ALD symptoms by restoring the production of metabolites, such as amino acids, in the intestine. Notwithstanding, further studies are needed to establish the relationship between the large intestine and severe ALDs (liver fibrosis and cirrhosis) or various organs (including the gut-liver, kidneys, and muscle) for a better understanding of the pathological changes induced by alcohol consumption.

## Data availability statement

The datasets presented in this study can be found in online repositories. The names of the repository/repositories and accession number(s) can be found in the article/Supplementary material.

## Ethics statement

All animal procedures were performed according to the National Institutes of Health Guidelines for the Humane Treatment of Animals, with approval from the Institutional Animal Care and Use Committee of the World Institute of Kimchi (WIKIM IACUC 201936). Animals were sacrificed *via* forced CO2 inhalation and all efforts were made to minimize suffering.

## Author contributions

SHL, BR, NH, H-JC, and SKL contributed to the conception and design of the study. JK, SA, and SHL performed the experiments and wrote the manuscript. JYK and TW performed the bioinformatic and statistical analysis. TW and SR revised the entire manuscript. All authors contributed to the article and have approved the submitted version.
