# Supplementary material for: Probiotic Lactobacilli ameliorate alcohol-induced hepatic damage via gut microbial alteration
Source: Front Microbiol. 2022 Aug 18;13:869250. doi: 10.3389/fmicb.2022.869250 (PMC9446534; doi:10.3389/fmicb.2022.869250)
Supplement: Supplementary file 1 [file Data_Sheet_1.PDF]

Supplementary Material

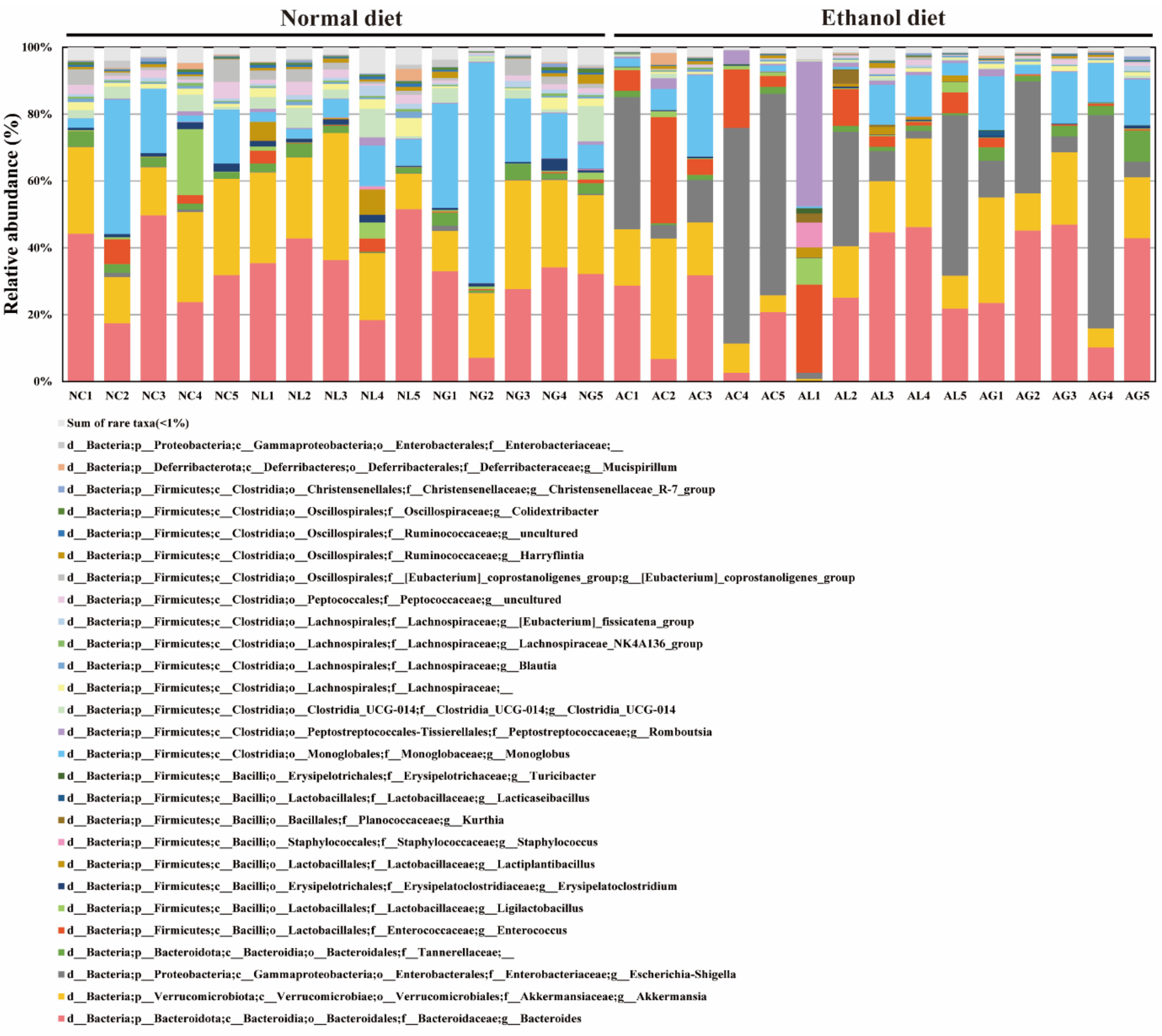

Supplementary Figure S1. Effect of administering kimchi-derived LAB and *Lacticaseibacillus rhamnosus* GG on the gut microbial community in mice fed on normal and alcohol diet. The relative abundance of the gut microbiota was determined at the genus level. The relative bacterial mean abundances were calculated by the mean values of relative phylotypic compositions of respective fecal samples. The sum of rare taxa consisted of genera showing a percentage of reads < 1% of the

total reads in all subjects. NL, normal diet and *Lactiplantibacillus plantarum* DSR J266 + *Lactobacillus brevis* DSR J266 group; NG, normal diet and *Lacticaseibacillus rhamnosus* GG group; AC, alcohol diet group; AL, alcohol diet and *Lp. plantarum* DSR J266 + *Lv. brevis* DSR J301 group; AG, alcohol diet, and *Lb. rhamnosus* GG group.
