# Supplementary material for: Probiotic Lactobacilli ameliorate alcohol-induced hepatic damage via gut microbial alteration
Source: Front Microbiol. 2022 Aug 18;13:869250. doi: 10.3389/fmicb.2022.869250 (PMC9446534; doi:10.3389/fmicb.2022.869250)
Supplement: Supplementary file 2 [file Table_1.DOCX]

Supplementary Material

**Supplementary Table S1.** Overview of the mice gut bacterial 16S rRNA gene sequence dataset

| Sample-id | Input | Quality filtered | Denoised | Merged | Non-chimeric | Singleton-removed |
| --- | --- | --- | --- | --- | --- | --- |
| NC1 | 122335 | 95111 | 93248 | 89001 | 70436 | 70436 |
| NC2 | 138609 | 105144 | 103499 | 100342 | 80546 | 80540 |
| NC3 | 109214 | 87607 | 85840 | 81657 | 58622 | 58621 |
| NC4 | 99629 | 77783 | 75306 | 71063 | 53274 | 53273 |
| NC5 | 112607 | 87896 | 86263 | 83244 | 61442 | 61442 |
| NL1 | 133517 | 105497 | 102975 | 97334 | 74902 | 74900 |
| NL2 | 106135 | 84984 | 82854 | 78987 | 59844 | 59841 |
| NL3 | 106766 | 82007 | 80404 | 77135 | 60890 | 60890 |
| NL4 | 155594 | 122184 | 119553 | 113191 | 87289 | 87289 |
| NL5 | 134522 | 104434 | 102496 | 97774 | 78691 | 78686 |
| NG1 | 104032 | 81530 | 79475 | 75350 | 49938 | 49938 |
| NG2 | 104855 | 80000 | 79273 | 78152 | 65167 | 65167 |
| NG3 | 129030 | 100041 | 98608 | 95449 | 72696 | 72696 |
| NG4 | 125786 | 93751 | 91545 | 86769 | 67352 | 67352 |
| NG5 | 139835 | 109706 | 107002 | 101525 | 78173 | 78168 |
| AC1 | 128254 | 102219 | 101579 | 100193 | 90591 | 90571 |
| AC2 | 125546 | 98111 | 96939 | 95008 | 79404 | 79396 |
| AC3 | 123955 | 97966 | 96208 | 92723 | 69474 | 69470 |
| AC4 | 115962 | 92199 | 91843 | 90570 | 76199 | 76196 |
| AC5 | 155157 | 123820 | 122796 | 120400 | 107284 | 107269 |
| AL1 | 169708 | 135532 | 134681 | 130883 | 86070 | 86070 |
| AL2 | 130262 | 105624 | 104931 | 103107 | 90940 | 90896 |
| AL3 | 143353 | 112997 | 111160 | 106813 | 82390 | 82389 |
| AL4 | 117481 | 91596 | 90350 | 87262 | 69666 | 69666 |
| AL5 | 109262 | 87205 | 85909 | 82475 | 61835 | 61835 |
| AG1 | 123680 | 96193 | 94682 | 91316 | 73640 | 73636 |
| AG2 | 133451 | 109344 | 108091 | 105521 | 89158 | 89156 |
| AG3 | 134393 | 107404 | 106462 | 104378 | 90428 | 90417 |
| AG4 | 127879 | 101438 | 100800 | 99288 | 91526 | 91504 |
| AG5 | 116160 | 90669 | 89257 | 85946 | 62382 | 62382 |
